# A Prospective Randomized Double-Blind Study Evaluating UP165 and *S*-Adenosyl-l-Methionine on Depression, Anxiety and Psychological Well-Being

**DOI:** 10.3390/foods4020130

**Published:** 2015-05-08

**Authors:** Douglas S. Kalman, Samantha Feldman, Rafeal Rivas Vazquez, Diane R. Krieger

**Affiliations:** 1 Miami Research Associates, Endocrinology and Nutrition Department, 6141 Sunset Drive, Suite 301, Miami, FL 33143, USA; E-Mails: Samantha.Feldman@qps.com (S.F.); diane.krieger@qps.com (D.R.K.); 2 Miami Research Associates, Neurosciences Department, 6141 Sunset Drive, Suite 301, Miami, FL 33143, USA; E-Mail: Rafael.Rivas-vazquez@qps.com

**Keywords:** depression, anxiety, dietary supplement, *S*-adenosyl-methionine(SAM-e), *Zea mays* L.

## Abstract

The primary objective of this pilot clinical trial was to evaluate the effects of UP165 (derived from *Zea mays* L., commonly known as corn) over time. The secondary objective was the comparison for outcomes *versus* *S*-adenosyl-methionine (SAM-e). Subjects with mild depression or anxiety were given the Beck Depression Inventory second edition (BDI-II), the Beck Anxiety Inventory (BAI), and the Schwartz Outcome Scale (SOS-10). Forty-two subjects (21–65 years old) were randomized to eight-weeks of supplementation with UP165 or SAM-e with questionnaires being administered at randomization, week four and eight. Those receiving UP165 achieved significant reduction from baseline at weeks four and eight, respectively for the BDI-II, as well as a trend for reduction in BAI at week four and significance at week eight. There was a trend for improvement on the SOS at week four and significance at week eight. SAM-e demonstrated a trend for improvement on the BDI-II by week eight over the UP165 with no differences between the two for the BAI or the SOS. Overall, this study indicates that there may be benefit to UP165 for mood enhancement in those with mild depression or anxiety. Randomized placebo comparator trials appear warranted.

## 1. Introduction

Depression affects approximately 20.9 million Americans every year and costs an estimated $34 billion in direct and indirect expenses. Major depression is a common, serious, recurrent disorder linked to diminished daily functioning and quality of life. It is associated with increased medical morbidity, and mortality. The World Health Organization (WHO) has ranked depression as the fourth leading cause of disability worldwide and projects that, by 2020, it will be the second leading cause [1,2]. While anxiety is clinically defined as a separate disorder, depression and anxiety disorders overlap considerably with 50%–60% of depressed individuals meeting lifetime criteria for anxiety disorders Furthermore, there is significant overlap in diagnostic criteria and risk factors for the disorders. Therefore, it is acceptable not to differentiate them [3]. In addition to the various traditional pharmaceutical products that are available for antidepressant therapy, there is an interest in identifying complementary and alternative medicines (CAM) that may demonstrate mood enhancing effects and be cost effective [4]. CAM therapies are widely used by consumers to treat depression, in fact, 41% of severely depressed individuals use at least one CAM therapy, compared to 28% of the healthy population. Furthermore, 9% of severely depressed use only CAM therapy [5]. To date, various nutritional supplements (e.g., folate, St. John’s Wort) show some degree of efficacy in treating mild mood disorders [6]. One of the best studied and most widely used of these supplements is *S*-adenosyl-methionine (SAM-e), a naturally occurring compound in the body that has demonstrated promising efficacy in the treatment of depression and anxiety [6]. There is extensive research to support its efficacy and safety for treating mild to moderate depression [5,7]. It has even been shown to complement traditional pharmaceutical treatment. Significant improvement in depression scores were shown in patients on Selective Serotonin Reuptake Inhibitors (SSRIs) taking 1600 mg/day of SAM-e compared to those taking a placebo [8].

SAM-e is essential for three metabolic pathways that stimulate more than 100 biochemical reactions. One of these pathways, transmethylation (methyl donor), is known to influence levels of biogenic amine neurotransmitters (e.g., serotonin, dopamine, norepinephrine). It is postulated that SAM-e exerts its mood enhancing effects through this transmethylation mechanism [9].

UP165, a compound derived from *Zea mays* L. (commonly known as corn), which contains the naturally occurring substance 6-methoxybenzoxazolinone (6-MBOA). 6-MBOA has a structural resemblance to melatonin [10], and has demonstrated various biological effects, such as progonadal properties, antifungal effects, and antiviral activity [11,12,13,14,15]. Furthermore, 6-MBOA stimulates melatonin biosynthesis [16]. 6-MBOA appears to act as a weak β-adrenergic agonist and seems to have affinity for melatonin receptors [17]. It is also well established that serotonin is a precursor of melatonin [18]. In light of the structural and biological similarities between 6-MBOA and melatonin, there is reason to suspect that 6-MBOA may demonstrate mood enhancing properties [19,20].

The primary objective of this prospective pilot study was to determine whether UP165 had efficacy in enhancing psychological well-being and in relieving symptoms of depression and anxiety, when compared to baseline scores with a secondary comparative objective *versus* SAM-e.

## 2. Experimental Section

A prospective, randomized, double-blind, 8-week clinical trial was conducted at Miami Research Associates in Miami, FL, USA. The study was approved by Aspire Institutional Review Board (project identification code SEROC-2006, approval date 6 September 2006). The study sample consisted of 42 male and female subjects aged 21 to 65 years old recruited through advertisement who provided informed consent. Subjects were included if they were found to have mild to moderate levels of depression and anxiety as evidenced by the Beck Depression Inventory-II (BDI-II) (scores of 10–19) and the Beck Anxiety Inventory (BAI) (scores of 10–21). The BDI-II has been used in over 7000 studies. It has been studied in multiple clinical and non-clinical populations and shown to demonstrate high reliability, capacity to discriminate between depressed and non-depressed subjects, and improved concurrent, content, and structural validity [21]. Subjects were excluded if more severe levels of depression (e.g., >19 on the BDI-II) or anxiety (e.g., >21 on the BAI) were detected, or if the subjects presented a history of bipolar disorder, psychosis or other severe psychiatric disorders. Subjects were excluded if they took any HMG-CoA reductase inhibitors (“statins”) or medications for depression and anxiety within the past year (unless if it was for less than one-month) and if at the time of screening suffered from any cardiovascular, neurological, hepatic, thyroid disorders, or Type I and II diabetes, or had a history of cancer within five years of screening. Subjects unable to understand the informed consent, those with corn allergies or currently pregnant, breastfeeding, or planning on becoming pregnant during the study were also excluded. Forty-two subjects were enrolled in the study with 34 subjects included in the efficacy analysis. Eight subjects were excluded from efficacy analysis due to two subjects not completing all the scheduled visits and three subjects having insufficient product compliance (UP165 *n* = 19 and SAM-e *n* = 15). Three subjects were lost to follow-up and not included in the safety or efficacy analyses. Subjects were randomized to receive a dietary supplement of either UP165 (derived from *Zea mays* L.) 250 mg/day or SAM-e 400 mg/day. SAM-e (an active comparator) was chosen (this is the common commercially sold dosage) opposed to a placebo due to high suicide rate and suicidal ideation in patients with untreated depression [5]. Baseline demographics and characteristics are noted in Table 1.

Subjects’ scores of depression and anxiety as measured by the BDI-II and BAI were assessed at visits 1, 3, and 4. Subjects’ wellbeing was assessed using the Schwartz Outcome Scale (SOS-10). Scores of wellbeing were measured at visits 2, 3, and 4. Safety analysis included comprehensive metabolic panel, complete blood count with differential (Quest Diagnostics, Miramar, FL, USA) and monitoring of blood pressure and heart rate.

Adverse events (AE) were listed, MedDRA encoded, grouped by general type of event (gastrointestinal, neurological, cardiac, *etc*.), and cross tabulated by event type and product. Differences in patterns of AE between products were tested by the Fisher Exact test.

Statistical controls and methodology included testing the data for normality (Anderson-Darling test) and if non-normal distribution, the data was analyzed by non-parametric methods. Changes in quantitative data were analyzed by the paired *T*-test or Wilcoxon if non-parametric. Categorical data was analyzed over the course of intervention by the Fisher Exact Test. Secondary analyses for comparing changes between the two interventions was by the Student *T*-test or Mann-Whitney if non-parametric. If there were any subject characteristics that were significantly different at baseline, these were treated as covariates and a general linear mixed model was employed. A power and sample size calculation allowed for 20 subjects per group in order to detect an effect size of 0.67 sigma (slightly larger than a medium effect by Cohen’s convention), thus, power was set at 80%. If there were missing values or data on an efficacy variable, the last observation carried forward (LOCF) imputation was utilized. Type I error was controlled by employing a hierarchical testing approach with statistical significance at *p* < 0.05. Data entry was on Microsoft Excel (2003) and all data analysis was performed using the “***R***” Program v2.5.1 (***R*** Foundation for Statistical Computing, Vienna, Austria, www.R-project.org).

## 3. Results

Of the 34 subjects included in the efficacy analysis 85% (*n* = 29) were female and 15% (*n* = 5) were male, with a mean age of 39.2 ± 11.0 years. Subjects were randomized to consume either UP165 (*n* = 19) or SAM-e (*n* = 15). There were no significant differences between both groups in regards to baseline characteristics including BDI-II and BAI scores at screening. All subjects reported signs of mild depression at baseline, while at week eight subjects reported scores of minimal depression. No serious adverse events or clinically relevant changes in vital signs were noted during this trial. All AE that did occur were coded as mild and most likely not related to the products. No significant differences in the incidence of AE or their distribution across organ systems were observed. No product safety concerns were raised from this study.

As the primary objective of the study was to determine the effects of intervention with UP165 on mood states, intervention with UP165 was shown to reduce the BDI-II by greater than 50% from baseline to week four (17.2 ± 4.2 to 10.2 ± 7.2; *p* < 0.001) and this reduction remained significant at week eight (17.2 ± 4.2 to 11.8 ± 8.4; *p* < 0.012). UP165 was found to have a significant effect on anxiety at the four-week mark (12.8 ± 5.4 to 6.7 ± 5.6; *p* < 0.001), which remained significant at week eight (12.8 ± 5.4 to 8.1 ± 7.4; *p* = 0.01). Data is shared in Table 1, Table 2 and Table 3. UP165 was not found to have an effect on the SOS-10 scores (see Table 4).

Intervention with SAM-e also led to significant reduction in the BDI-II scores at weeks four and eight, respectively (16.1 ± 3.5 to 9.0 ± 7.0; *p* = 0.001 and 16.1 ± 3.5 to 7.2 ± 7.4; *p* = 0.002). SAM-e also had significant effects as measured by the BAI at week eight (12.9 ± 6.4 to 6.9 ± 5.6; *p* = 0.026). Intervention with SAM-e produced significant changes in the SOS-10 score at week eight (37.1 ± 9.7 to 41.2 ± 13.7; *p* = 0.038) (Table 1, Table 2, Table 3 and Table 4).

Comparative data was included as part of the secondary objective of the study. At week eight, the difference between UP165 and SAM-e trended for significance (11.8 ± 8.4 *vs.* 7.2 ± 7.4; *p* = 0.063). There were no other significance differences between the interventions on the outcomes observed.

**Table 1:** Baseline demographics and characteristics.

Variable	SAM-e	UP165	*p*-Value
Age	44.0 ± 12.7 (21)	38.4 ± 10.1 (21)	0.121
years	46 (22–64)	36 (22–63)	
Gender			
Female	17 (81%)	17 (81%)	1.000
Male	4 (19%)	4 (19%)	
Total	21 (100%)	21 (100%)	
Height	162.0 ± 7.9 (20)	164.1 ± 7.7 (21)	0.396
cm	161.9 (150.4–179.6)	161.4 (150.4–181)	
Body Weight	73.8 ± 20.1 (20)	75.9 ± 20.4 (21)	0.648
kg	67.7 (37.8–120.3)	67 (47.8–127.6)	
BDI-II: Beck Depression	17.3 ± 4.0 (21)	17.2 ± 4.0 (21)	0.909
Inventory, Version II	17 (12–26)	17 (9–25)	
BAI: Beck Anxiety	12.8 ± 6.7 (21)	13.4 ± 5.5 (21)	0.727
Inventory	11 (3–26)	11 (3–22)	
SOS-10: Schwartz	40.0 ± 10.7 (21)	38.8 ± 9.0 (21)	0.711
Outcome Scale	38 (17–59)	39 (25–59)	

Data is reported as: mean ± standard deviation (number of subjects); mean (range); *p* < 0.05 significant.

**Table 2:** BDI-II: Beck Depression Inventory.

Visit	SAM-e	UP165	*p*-Value
Visit 1	16.1 ± 3.5 (15)	17.2 ± 4.2 (19)	0.427
Screening	15 (12–23)	17 (9–25)	
Visit 3	9.0 ± 7.0 (15)	10.2 ± 7.2 (19)	0.424
Week 4	6 (1–22)	8 (0–28)	
Visit 4	7.2 ± 7.4 (15)	11.8 ± 8.4 (19)	0.063
Week 8	5 (0–27)	10 (0–30)	
Change	−7.1 ± 6.9 (15)	−7.1 ± 5.7 (19)	0.971
from Baseline	−9 (−18–6)	−7 (−16–6)	
to Week 4	*p* = 0.001	*p* < 0.001	
Change	−8.9 ± 6.9 (15)	−5.4 ± 7.3 (19)	0.055
from Baseline	−10 (−16 – 6)	−7 (−18–12)	
to Week 8	*p* = 0.002	*p* = 0.012	

Data is reported as: mean ± standard deviation (number of subjects), mean (range), *p* value within row is significance within group, *p* value in its own column is between groups, *p* < 0.05 is considered significant.

**Table 3:** BAI: Beck Anxiety Inventory.

Visit	SAM-e	UP165	*p*-Value
Visit 1	12.9 ± 6.4 (15)	12.8 ± 5.4 (19)	0.970
Screening	11 (3–25)	11 (3–21)	
Visit 3	8.1 ± 6.6 (15)	6.7 ± 5.6 (19)	0.464
Week 4	7 (1–24)	4 (1–18)	
Visit 4	6.9 ± 5.6 (15)	8.1 ± 7.4 (19)	0.862
Week 8	7 (0–22)	6 (1–30)	
Change	−4.8 ± 10.3 (15)	−6.1 ± 5.2 (19)	0.647
from Baseline	−6 (−22–13)	−4 (−16–1)	
to Week 4	*p* = 0.092	*p* < 0.001	
Change	−6.0 ± 9.4 (15)	−4.7 ± 7.1 (19)	0.644
from Baseline	−2 (−24–11)	−6 (−16–11)	
to Week 8	*p* = 0.026	*p* = 0.010	

Data is reported as: mean ± standard deviation (number of subjects), mean (range), *p* value within row is significance within group, *p* value in its own column is between groups, *p* < 0.05 is considered significant.

**Table 4:** SOS-10: Schwartz Outcome Scale.

Visit	SAM-e	UP165	*p*-Value
Visit 1	37.1 ± 9.7 (15)	38.0 ± 8.1 (19)	0.777
Screening	36 (17–55)	39 (25–56)	
Visit 3	40.5 ± 11.3 (15)	39.4 ± 13.7 (19)	0.804
Week 4	41 (11–59)	40 (11–59)	
Visit 4	41.2 ± 13.7 (15)	36.9 ± 16.4 (19)	0.742
Week 8	43 (10–60)	46 (10–54)	
Change	3.3 ± 6.8 (15)	1.4 ± 12.3 (19)	0.584
from Baseline	4 (−8–19)	3 (−25–22)	
to Week 4	*p* = 0.077	*p* = 0.635	
Change	4.1 ± 6.6 (15)	−1.1 ± 17.5 (19)	0.755
from Baseline	6 (−8–12)	5 (−39–22)	
to Week 8	*p* = 0.038	*p* = 0.952	

Data is reported as: mean ± standard deviation (number of subjects), mean (range), *p* value within row is significance within group, *p* value in its own column is between groups, *p* < 0.05 is considered significant.

## 4. Discussion

The present study was the first clinical trial to examine the efficacy of UP165 on mood-enhancing properties in humans. UP165 was found to have a positive effect on mood states (Beck Depression Inventory vII). The BDI-II contains 21 items, which assess depressive symptoms on a Likert-Scale. Clinical interpretation of scores is accomplished through criterion-referenced procedures. A 50% reduction in the BDI-II scores is considered a clinically meaningful change (personal communication, Aaron T. Beck, MD). The evidence in this pilot clinical trial indicates that more robust research for this potential natural intervention for mood disturbances is warranted. UP165 is known to have structural similarity to melatonin. Melatonin is known to affect mood states. In fact, melatonin is used as a biomarker for depression, and a direct relationship between melatonin and serotonin has recently been discussed [18]. It is also well established that serotonin is a precursor of melatonin and that melatonin disturbances have been linked to seasonal affective disorder and other forms mild depression. There is evidence suggesting that improving Melatonin levels improves mood and mild depression [21,22]. Therefore, as 6-MBOA (UP165) has melatonin-like effects and is structurally similar, the effects on mood might be mediated by the interaction with the hypothalamic-pituitary-adrenocortical (HPA) system. The mechanism of action may also be related to the structure of this novel food compound. 6-Methoxy-2-benzoxazolinontr-(6-MBOA: UP165), is a structural analogue of melatonin (aMT) that has been shown to inhibit tryptophan 2,3-dioxygenase activity in both the photo-phase (light-phase) and the scoto-phase (circadian rhythms) with greater potency during the light-phase. Further, studies have evaluated the effects of 6-MBOA on the brain tryptophan hydroxylase (TH) activity, which is a rate-limiting enzyme in serotonin (5-HT) biosynthesis and subsequently on serotonin levels. The findings showed that, 6-MBOA induces TH activity with a concomitant rise in brain serotonin levels [23,24]. Therefore, it is possible that within this pilot trial, the UP165 had a positive impact on mood states by modulating serotonin levels, although this was not directly assessed. Considering that melatonin has been shown to play a role in regulating sleep cycles and circadium rhythm, it is possible that the people taking UP165 experienced better sleep quality and therefore better mood state [22,23,24,25]. However, we did not measure sleep quality.

Mood states as examined by the Beck Anxiety Inventory (BAI) also demonstrated that intervention with UP165 had an effect. The BAI reliably discriminates anxiety from depression while displaying convergent validity. The scale, consisting of 21 items that describe a common symptom of anxiety, asks respondents to rate how much s/he has been bothered by each symptom utilizing a point scale ranging from 0 to 3. Those on UP165 experienced a reduction in BAI scores consistent with lessened anxiety. This effect was significant throughout the study whereas SAM-e intervention appeared to take longer to have an effect (only significant at week eight). Prior studies have not found a ubiquitous effect of SAM-e for anxiety, in fact, anxiety has been observed as a side effect in some of the clinical trials [4,6]. The apparent efficacy of UP165 for mild anxiety might also be due to any effects it may have on serotonin levels. Further research is necessary to determine the mechanism of action of UP165 on anxiety.

The Schwartz Outcome Scale (SOS-10) is a validated tool for measuring and assessing physical, emotional, and psychological perceptions of health and wellbeing. The scale consists of 10 questions, each using a 7-point Likert scale ranging from 0 (never) to 6 (always). The SAM-e group showed a significant mean increase (improvement) on the SOS-10 scale while no significant change over time was observed for UP165. There were no between group differences on this psychometric marker of health and wellbeing.

Future research is also necessary to better discriminate the psychotropic effects of UP165 by utilizing a placebo arm. In the current study both groups improved over time in many of the study parameters and it is wholly possible that this was due to either a Hawthorne effect or a mass placebo response. However, since SAM-e has been established as a nutritional intervention for mood disorders, using it as a benchmark for a novel new nutritional compound (UP165) appears to have merit [4,6,9,26]. The effectiveness of UP165 within this pilot early phase clinical trial mirrors that of the early published experience with SAM-e [27]. Further, an authoritative review found that SAM-e was safe and effective for the intervention of depression (equal to that of tri-cyclic medications) [27]. The early evidence for how UP165 may affect mood states in those with mild depression or anxiety appears to be worthwhile for further research and development of this naturally occurring food compound. In addition, the strength of future research can be increased by having a randomized double blind placebo controlled clinical trial. 

## 5. Conclusions

UP165 shows promise as a nutritional psychotropic intervention for enhancing mood states. 
